# Improving Diagnostic Accuracy for Surgical Pelvic Organ Prolapse: A Sequential Protocol Combining POP-Q Examination and Transperineal Ultrasound

**DOI:** 10.3390/jcm15030979

**Published:** 2026-01-26

**Authors:** José Antonio García-Mejido, Ana Hurtado-Guijosa, Ana Fernández-Palacín, Fernando Fernández-Palacín, Fernando Bugatto, José Antonio Sainz-Bueno

**Affiliations:** 1 Department of Surgery, Faculty of Medicine, University of Seville, 41014 Seville, Spain; anahurtado96@hotmail.com (A.H.-G.); jsainz@us.es (J.A.S.-B.); 2 Biostatistics Unit, Department of Preventive Medicine and Public Health, University of Seville, 41014 Seville, Spain; afp@us.es; 3 Department of Statistics and Operational Research, University of Cádiz, 11003 Cádiz, Spain; fernando.fernandez@uca.es; 4 Department of Child and Mother Health and Radiology, School of Medicine, University of Cádiz, 11009 Cádiz, Spain; fernando.bugatto@gm.uca.es; 5 Division of Maternal-Fetal Medicine, Obstetrics and Gynecology Department, Puerta Del Mar University Hospital, Biomedical Research and Innovation Institute of Cádiz (INiBICA), 11009 Cádiz, Spain

**Keywords:** pelvic organ prolapse, POP-Q system, ultrasonography, transperineal ultrasound, gynecologic surgery, diagnostic protocol, urogynecology, surgical planning

## Abstract

**Background/Objectives:** The POP-Q system is conventionally used to evaluate pelvic organ prolapse (POP). Nevertheless, differences between clinical examination and intraoperative findings can hinder appropriate surgical planning. We aimed to assess the accuracy of a sequential protocol involving clinical POP-Q assessment and, in cases of uncertain diagnosis, transperineal ultrasound. **Methods:** We conducted a prospective observational study with 314 women scheduled for POP surgery from January 2021 to December 2024. A pelvic floor specialist assessed all patients using the POP-Q system. Transperineal ultrasound was carried out only when the clinical diagnosis remained uncertain. We compared the accuracy of this sequential approach (POP-Q ± ultrasound) versus POP-Q alone, using intraoperative findings as the gold standard. Sensitivity and specificity were determined for each type of prolapse. **Results:** Of the 314 patients, 181 (57.6%) had a definitive diagnosis with POP-Q alone, whereas 133 (42.4%) required additional ultrasound. In these uncertain cases, the addition of ultrasound significantly increased sensitivity for cystocele (by 0.5–11.3%), uterine prolapse (45.5–63.7%), cervical elongation (5.2–21.4%), rectocele (5.7–16.4%), and enterocele (58.7–74.7%) (all *p* < 0.05). Specificity also improved for uterine prolapse, cervical elongation, and rectocele. The sequential protocol’s performance in uncertain cases was comparable to clinical examination in straightforward cases. **Conclusions:** Applying a sequential protocol that adds transperineal ultrasound for unclear cases significantly increases diagnostic precision for surgical POP, potentially optimizing surgical planning.

## 1. Introduction

Pelvic organ prolapse (POP) is an increasingly common condition in our setting. The lifetime risk of undergoing POP surgery is 11% [1], which corresponds to over 500,000 reconstructive surgeries annually in the United States [2,3]. The standard diagnostic method before POP treatment to objectively assess the extent of prolapse is clinical examination based on the pelvic organ prolapse quantification (POP-Q) system [4], which is the most widely used system [5,6]. The POP-Q system is objective and specific for describing and staging POP [7]. However, it was initially described that its intraobserver and interobserver reliability was poorer for the central compartment [8]. Additionally, prolapses in the central and posterior compartments were found to be more pronounced on intraoperative examination [9]. In fact, it is agreed that the examination in the operating room, with the patient under anesthesia, differs from the preoperative examination in the consultation [10].

Historically, in our hospital, we performed surgery on POP patients based solely on the preoperative indication made during the consultation. This approach caused discomfort among surgeons, as they felt that the POP was different between the preoperative examination and intraoperative findings, as previously described in the literature [8]. Subsequently, the development of transperineal pelvic floor ultrasound demonstrated several advantages over POP-Q system-based examination, as it used a fixed reference point (posteroinferior edge of the pubic symphysis) [11,12,13,14], controlled confounding factors influencing the diagnosis of POP (such as bladder filling) [15], assessed coactivation of the levator ani muscle during Valsalva [16], and evaluated the duration of the Valsalva maneuver [17]. Furthermore, transperineal ultrasound has shown good diagnostic capacity for POP with sensitivity ranging from 60% to 93% and specificity from 64% to 95% [11,12,13,14]. For these reasons, we decided to systematically include transperineal ultrasound in consultation to assist the surgeon in diagnosing surgical POP. This ultrasound is performed in cases of diagnostic uncertainty. We believe that the incorporation of transperineal ultrasound for the evaluation of surgical POP in our center has influenced the types of surgical procedures performed on our patients with surgical POP. Accurate preoperative assessment is not merely academic; it is critical for surgical planning. A discrepancy between the clinical examination and the actual prolapse found during surgery can lead to unmasked defects being left untreated or, conversely, to unnecessary procedures. As highlighted in previous studies [7,9], reliance solely on physical examination may underestimate the extent of apical or high-grade prolapse, potentially contributing to the risk of surgical failure or recurrence. Therefore, our objective is to assess the impact of a sequential diagnostic protocol for surgical POP based on the introduction of transperineal ultrasound on preoperative clinical examination to establish the optimal corrective surgery for POP.

## 2. Materials and Methods

A prospective observational study was conducted, recruiting all patients (*n*: 314) undergoing surgical correction of pelvic organ prolapse (POP) between 1 January 2021, and 31 December 2024. The study was approved by Biomedical Ethics Committee of the Junta de Andalucía (1259-N-20) and conducted in accordance with the Declaration of Helsinki (as revised in 2013).

Women who had previously undergone pelvic floor repair were not eligible for inclusion. Data collection comprised maternal age, menopausal state, body mass index (BMI), and obstetric background (including number of deliveries, cesarean sections, and miscarriages). Additionally, the specific type and severity of prolapse were documented, categorizing cases as cystocele, uterine descent, isolated cervical elongation, rectocele, or enterocele.

**Figure 1:**
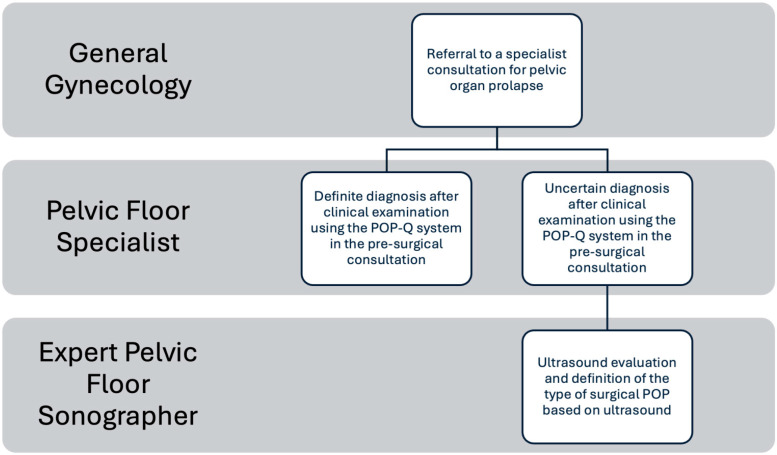
The sequential protocol for the diagnosis of surgical POP.

The sequential diagnostic protocol (Figure 1) commenced with a standardized clinical examination using the POP-Q system for all patients. This assessment was conducted by a senior gynecologist with specialized expertise in pelvic floor pathology (>20 years of experience).

Based on this initial assessment, the study population was stratified into two groups. We defined diagnostic uncertainty as cases with discordance between symptoms and physical examination (e.g., sensation of lump vs. low-stage POP-Q), or when the specific compartment (e.g., high rectocele vs. enterocele) was unclear on POP-Q due to anatomical exposure.

These specific cases of diagnostic uncertainty were deliberated within a multidisciplinary pelvic floor board, whereupon transperineal ultrasound was proposed as a complementary modality to acquire additional objective data.

### 2.1. Management of Patients with a Definite Diagnosis After Clinical Examination Using the POP-Q System in the Preoperative Consultation

Patients referred to the pelvic floor consultation for POP were evaluated by a specialist in obstetrics and gynecology, with expertise in pelvic floor dysfunctions. All patients underwent a standardized interview along with a gynecological examination using the POP-Q system to define the type of POP [10]. Patients with symptomatic POP (stage 2 or greater) and previous failure of conservative treatment were considered candidates for surgery.

If the specialist established a definite diagnosis after clinical examination using the POP-Q system in the preoperative consultation, the type of surgical intervention was determined without the need for POP ultrasound (Figure 1).

### 2.2. Management of Patients with a Specific Diagnostic Uncertainty After Clinical Examination

For this group, a transperineal ultrasound was requested (Figure 1).

**Figure 2:**
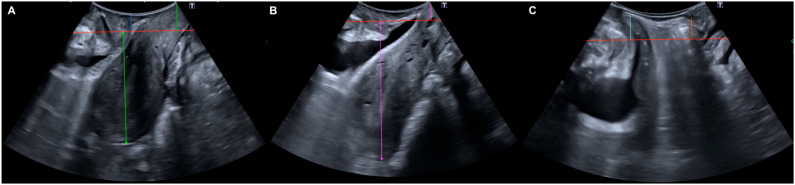
The three images display Valsalva ultrasound images in the mid-sagittal plane. The red line represents the posterior-inferior border of the pubic symphysis. (**A**): Cystocele: Descended urinary bladder by 10 mm or more (blue line). Uterine prolapse: Difference between resting and Valsalva of the pubis-uterine fundus distances of 15 mm or more (green arrow) and cervix descended by 15 mm or more (green line). (**B**): Cervical elongation without uterine prolapse: Difference between resting and Valsalva of the pubis-uterine fundus distances of less than 15 mm (pink arrow), with cervix descended by 15 mm or more (pink line). (**C**): Rectocele: Descended rectum by 15 mm or more (orange line) and enterocele: Descended enterocele region by 15 mm or more (light blue line).

Transperineal ultrasound assessments were conducted by experts in pelvic floor imaging who were blinded to the patients’ clinical data. A single examiner (J.A.G.M.) with >10 years of experience in pelvic floor imaging performed all ultrasounds. Surgical assessment served as the independent reference standard to minimize incorporation bias; intraoperative findings determined the final diagnosis regardless of imaging prediction. We employed a Toshiba^®^ 700 Aplio system (Toshiba Medical Systems Corp., Tokyo, Japan) equipped with a PVT-675 MV 3D abdominal transducer, which was sheathed in a sterile glove for hygiene. Imaging was performed with the patient in the dorsal lithotomy position, ensuring minimal transducer pressure on the perineum. The sonographic criteria used to diagnose the different types of POP were defined as follows: Cystocele: Descent of the bladder ≥ 10 mm below the posteroinferior margin of the pubic symphysis during maximal Valsalva [12,18]. Uterine Prolapse: A variation of ≥15 mm in the distance from the pubis to the uterine fundus between rest and Valsalva, accompanied by cervical descent of ≥15 mm [11,13]. Cervical Elongation (without uterine prolapse): Cervical descent ≥ 15 mm relative to the posteroinferior pubic margin during Valsalva, but with a change in the pubis-uterine fundus distance of <15 mm between resting and Valsalva states [11,13]. Rectocele: Rectal descent ≥ 15 mm relative to the posteroinferior pubic margin during Valsalva [12,18], evidencing protrusion of the anterior rectal wall into the vaginal lumen [19]. Enterocele: Descent of the enterocele pouch ≥ 15 mm relative to the posteroinferior pubic margin during Valsalva [12,18], characterized by the presence of abdominal contents anterior to the anorectal angle, creating a separation between the vagina and the rectal ampulla [19] (Figure 2).

### 2.3. Determination of the Gold Standard for Diagnosing Surgical POP

All patients underwent a clinical examination in the operating room, under epidural anesthesia, by the same expert specialist performing the surgical correction. This intraoperative assessment was designated as the diagnostic ‘gold standard’ for POP.

Crucially, the definitive surgical decision regarding which compartments to repair (all interventions utilized vaginal autologous tissue repair) was made by the same specialist. We performed standardized anterior and/or posterior colporrhaphy using native tissue with 2-0 absorbable polyglactin sutures. Apical fixation (sacrospinous ligament fixation or McCall culdoplasty) was added when indicated. This decision was informed by an integration of all available data: the initial preoperative POP-Q examination findings, the transperineal ultrasound results (where applicable), and the intraoperative examination findings. The specialist indicated and performed surgical correction of any compartment with a descent of stage II or greater.

### 2.4. Statistical Analysis

Numerical variables are summarized as means and standard deviations, while qualitative variables are summarized as frequencies and percentages. This analysis was performed for the two defined groups (definite diagnosis after clinical examination using the POP-Q system and uncertain diagnosis after clinical examination using the POP-Q system). To compare quantitative variables between the two study groups, the parametric Student’s T-test or the non-parametric Mann–Whitney U-test was used, depending on data normality (Shapiro–Wilk test). Chi-squared tests, Fisher’s exact test, or non-asymptotic Monte Carlo methods were used to analyze relationships between qualitative variables and POP. Sensitivity and specificity were calculated for clinical examination based on the stage of prolapse according to the POP-Q system for the definite diagnosis after clinical examination using the POP-Q system and uncertain diagnosis after clinical examination using the POP-Q system groups, using the type of corrective surgery performed in each compartment as the gold standard. In the uncertain diagnosis after clinical examination using the POP-Q system group, sensitivity and specificity were also assessed for ultrasound examination in defining surgical POP, using the type of corrective surgery performed in each compartment as the gold standard. All results were accompanied by 95% confidence intervals (CIs). Data analysis was conducted using the IBM SPSS Statistics 28.0 software for Windows.

## 3. Results

A total of 314 women were included, of whom 181 presented a definite diagnosis after clinical examination using the POP-Q system in the preoperative consultation, and 133 presented an uncertain diagnosis after clinical examination using the POP-Q system in the preoperative consultation. This indicates that the pelvic floor specialist was uncertain about the surgical diagnosis of POP in 42% (133/314) of the cases when applying the POP-Q system in the preoperative consultation, and these cases required a transperineal ultrasound (Figure 1). The comparative general characteristics between both groups are shown in Table 1. There were differences between the groups in patient profiles and the type of POP assessed during the preoperative consultation based on the POP-Q system. The patients in the definite diagnosis after clinical examination using the POP-Q system group were older (62.8 ± 8.9 vs. 56.5 ± 10.1; *p* < 0.001) and had a higher number of children (2.8 ± 1.3 vs. 2.3 ± 1.0; *p*: 0.001). Additionally, it can be noted that the definite diagnosis after clinical examination using the POP-Q system group had a higher rate of presence of cystocele (79.6% vs. 65.4%; *p*: 0.006), with a higher rate of Stage II or greater (*p*: 0.009), and presence of uterine prolapse (43.6% vs. 25.6%; *p*: <0.001), with a higher rate of Stage II or greater (*p*: <0.001), compared to the uncertain diagnosis after clinical examination using the POP-Q system group. Conversely, the presence of cervical elongation without uterine prolapse was higher in the uncertain diagnosis after clinical examination using the POP-Q system group compared to the definite diagnosis after clinical examination using the POP-Q system group (11.6% vs. 62.4%; *p*: <0.001), with a higher rate of Stage II or greater (*p*: 0.012).

In Table 2, we can observe the diagnostic capability for the different types of POP in patients with a definite diagnosis after clinical examination using the POP-Q system in the preoperative consultation and for the different types of POP in patients with an uncertain diagnosis after clinical examination using the POP-Q system in the preoperative consultation. When the pelvic floor specialist establishes a definite diagnosis after clinical examination using the POP-Q system, the sensitivity ranges from 92.9% to 100%, and the specificity ranges from 90.2% to 100%, depending on the compartment studied (Table 2). However, the sensitivity of the pelvic floor specialist is reduced when establishing an uncertain diagnosis after clinical examination using the POP-Q system, with sensitivity ranging from 33.3% to 91.7%, being minimal in the diagnosis of enterocele (33.3%) and uterine prolapse (40.9%). In this group, specificity was also reduced in the cases of uterine prolapse (83.8%). On the other hand, the diagnostic capability of ultrasound to detect surgical POP in cases where the pelvic floor specialist established an uncertain diagnosis after clinical examination using the POP-Q system was quite good, with sensitivity ranging from 92.8% to 100%, and specificity ranging from 81.6% to 99.1%, depending on the compartment studied (Table 2).

Based on these premises and utilizing our sequential diagnostic protocol for surgical pelvic organ prolapse (POP), we observed a substantial improvement in diagnostic capacity compared to the use of the POP-Q system in isolation (Table 3). With the sequential protocol, we achieved an improvement in the sensitivity for the minimum and maximum values for cystocele by 0.5% and 11.3% (*p* < 0.05), for uterine prolapse by 45.5% and 63.7% (*p* < 0.05), for cervical elongation by 5.2% and 21.4% (*p* < 0.05), for rectocele by 5.7% and 16.4% (*p* < 0.05), and for enterocele by 58.7% and 74.7% (*p* < 0.05), respectively. Additionally, with the sequential protocol, we observed an improvement in specificity for the minimum and maximum values for the diagnosis of uterine prolapse by 8.8% and 21.8% (*p* < 0.05), for cervical elongation by 12.0% and 27.9% (*p* < 0.05), and for rectocele by 3.9% and 11.9% (*p* < 0.05), respectively.

## 4. Discussion

We have observed that in cases where the pelvic floor specialist established an uncertain diagnosis after clinical examination using the POP-Q system in the preoperative consultation, diagnostic capacity was reduced. In these cases, by applying transperineal pelvic floor ultrasound to define surgical POP, we obtained diagnostic capacity similar to that of the specialist when establishing a definite diagnosis after clinical examination using the POP-Q system. Thus, we propose our sequential protocol for the diagnosis of surgical POP with a first step in which the pelvic floor specialist establishes a definite diagnosis after clinical examination, and a second step where transperineal ultrasound is applied in cases where the pelvic floor specialist establishes an uncertain diagnosis after clinical examination. This sequential protocol provides a substantial improvement in diagnostic capacity (sensitivity and specificity) compared to the usual diagnostic method based solely on clinical examination centered on the POP-Q system.

Existing evidence indicates that preoperative POP-Q assessment frequently underestimates the severity of prolapse in the apical and posterior compartments when compared to intraoperative findings [9]. Consequently, the concordance between office-based and surgical evaluations for the apical compartment is notably low [20]. Although the precise mechanism for this discordance has not been fully elucidated, it is hypothesized that muscle relaxation induced by regional anesthesia may be a contributing factor [21]. Another study determined that most POP-Q measurements with the Valsalva maneuver preoperatively are more representative of the degree of prolapse than those observed under anesthesia in the operating room [22]. However, in this study, the Valsalva maneuver in the operating room could not be assessed because general anesthesia was used [22]. In our case, the assessment of POP with Valsalva in the operating room was feasible, as epidural anesthesia was used for the surgical treatment of the patient. Other authors have argued that preoperative POP-Q system scores showed significant differences when evaluated intraoperatively under spinal anesthesia with gentle traction [23,24]. Swenson et al. described that almost 50% of women with cystocele or rectocele, with normal apical support on consultation examination, presented cervical locations below the hymen when examined with intraoperative traction [25]. These authors argue that patients should be informed that the surgical plan may change depending on intraoperative findings [23,24,25]. A recent study has demonstrated that transperineal ultrasound has greater concordance than the preoperative clinical examination for detecting POP with surgical indications in the central compartment [26]. In our study, we observed that changes in the surgical plan established during the preoperative consultation occurred primarily in cases where the pelvic floor specialist established an uncertain diagnosis after clinical examination using the POP-Q system. These errors in the preoperative clinical diagnosis of POP can be corrected by performing a transperineal ultrasound to determine surgical POP, as observed in our study. Therefore, to achieve the highest diagnostic performance, we propose a sequential diagnostic protocol for surgical POP based on two steps. The first step involves the pelvic floor specialist defining the type of surgical intervention based on the clinical examination with the POP-Q system performed during the preoperative consultation. The second step, applied to patients with an uncertain diagnosis after clinical examination using the POP-Q system, involves performing transperineal ultrasound of the pelvic floor to determine the type of surgical POP. With this sequential protocol, we will achieve higher diagnostic rates for surgical POP compared to those established by clinical examination alone, without the need to alter the surgical plan according to intraoperative findings, as previously proposed [23,24,25,26].

Furthermore, while other imaging modalities such as dynamic magnetic resonance imaging provide detailed anatomical information, transperineal ultrasound offers specific advantages in terms of cost-effectiveness and accessibility. Unlike magnetic resonance imaging, transperineal ultrasound allows for a dynamic bedside assessment that is easier to perform and well-tolerated by patients [27]. Moreover, previous research has established that transperineal ultrasound is highly reproducible and provides a functional evaluation of the pelvic floor that correlates well with patient symptoms, making it an ideal complementary tool for routine surgical planning [28].

The primary strength of this study lies in its prospective design, which minimizes selection and recall biases and ensures a standardized data collection process. Additionally, the implementation of a structured, clinically applicable diagnostic protocol in a real-world setting enhances the translational value of our findings. Another key strength is the blinded interpretation of ultrasound studies by expert sonographers, which reduces the risk of observer bias and strengthens the validity of the diagnostic comparisons. Furthermore, the use of intraoperative findings as the gold standard adds robustness to the assessment of diagnostic accuracy, aligning our results with real surgical decision-making.

A central component of our methodology is this pragmatic sequential protocol, which reserves ultrasound for cases with specific diagnostic uncertainty identified during the expert POP-Q examination. This ‘uncertainty’ represents not a deficient exam, but rather a senior specialist’s acknowledgment of the inherent limitations of POP-Q in complex cases. The cornerstone of this approach is the multimodal integration (POP-Q, ultrasound, and intraoperative assessment) by the same expert surgeon, which we contend ensures high diagnostic precision and optimal surgical planning, as our concordance results reflect.

However, there are limitations to consider. This study was conducted in a single high-volume academic center, which may limit generalizability to other settings with different patient populations or institutional practices. Moreover, the sonographers involved were highly experienced, which may not reflect the diagnostic performance achievable in less specialized or lower-resource environments. As such, reproducibility of these results in community-based hospitals or settings without pelvic floor imaging expertise may be limited. Future multicenter studies with a broader range of providers and institutional characteristics are warranted to validate and refine the applicability of this protocol across diverse clinical settings. Finally, transperineal ultrasound is operator-dependent. The specific training required may limit the reproducibility of this protocol in centers without experienced sonographers. Furthermore, this study focused on anatomical diagnostic accuracy and did not evaluate patient-reported outcome measures or postoperative symptom relief correlation, which remains a subject for future research.

The findings of this study have important clinical implications for the diagnostic workup and surgical planning of pelvic organ prolapse. By introducing a sequential protocol that incorporates transperineal ultrasound only in patients with uncertain clinical diagnoses after POP-Q examination, we demonstrate a pragmatic and resource-conscious strategy that maximizes diagnostic accuracy while avoiding unnecessary imaging. This approach allows for a more precise definition of the prolapse compartments involved, particularly in complex or borderline cases, and facilitates targeted surgical interventions. As a result, clinicians can provide more informed counseling to patients regarding surgical expectations, risks, and outcomes, thereby enhancing shared decision-making and preoperative consent processes.

From a health systems perspective, the implementation of this protocol may reduce intraoperative surprises and unplanned surgical modifications, which are known to affect operating time, resource allocation, and patient satisfaction. Integrating ultrasound into the diagnostic algorithm may also decrease the reliance on intraoperative assessment for surgical decision-making, shifting critical diagnostic steps to the preoperative phase where multidisciplinary discussion and planning are more feasible.

In terms of research, our results underscore the need for further validation in larger, multicenter cohorts. Future studies should investigate the learning curve associated with transperineal ultrasound interpretation, explore the cost-effectiveness of selective imaging strategies, and evaluate whether this protocol influences long-term surgical outcomes such as recurrence, complications, or quality of life.

## 5. Conclusions

A sequential diagnostic protocol using POP-Q assessment followed by transperineal ultrasound in selected cases significantly improves diagnostic accuracy for surgical POP. This approach enhances preoperative planning, reduces diagnostic uncertainty, and may improve patient counseling and surgical outcomes.

## Figures and Tables

**Table 1:** Comparison of the general characteristics and preoperative clinical examination using the POP-Q system between patients with definite diagnosis after clinical examination using the POP-Q system and patients with uncertain diagnosis after clinical examination using the POP-Q system.

	Definite Diagnosis After Clinical Examination Using the POP-Q System (*n*:181)	Uncertain Diagnosis After Clinical Examination Using the POP-Q System (*n*:133)	*p*	IC 95%
Age	62.8 ± 8.9	56.5 ± 10.1	<0.001	4.11: 8.37
Menopause age	49.9 ± 3.9	50.5 ± 3.8	0.310	−0.001; 1.00
BMC	27.6 ± 4.5	27.8 ± 5.0	0.960	−1.04; 0.01
Obstetric history				
Births	2.8 ± 1.3	2.3 ± 1.0	0.001	−1.0; −0.001
Cesarean sections	0.06 ± 0.23	0.08 ± 0.27	0.475	−0.001; 0.001
Abortions	0.3 ± 0.7	0.4 ± 0.7	0.551	−0.001; 0.001
Presence of cystocele	144/181 (79.6%)	87/133 (65.4%)	0.006	4.1%; 24.0%
Stage of cystocele				
Stage I	5/144 (3.5%)	6/87 (6.9%)	0.009	−10.2%; 2.7%
Stage II	13/144 (9.0%)	19/87 (21.8%)		−22.8%; −3.0%
Stage III	126/144 (87.5%)	34/87 (71.3%)		5.3%; 27.1%
Presence of uterine prolapse	79/181 (43.6%)	34/133 (25.6%)	<0.001	7.5%; 28.1%
Stage of uterine prolapse				
Stage I	22/79(27.8%)	7/34 (20.6%)	<0.001	−10.6%; 22.9%
Stage II	5/79 (6.3%)	11/34 (32.4%)		−42.3%; −9.5%
Stage III	40/79 (50.6%)	16/34 (47.1%)		−16.2%; 23.0%
Stage IV	12/79 (15.2%)	0/34 (0%)		3.6%; 22.9%
Presence of cervical elongation without uterine prolapse	21/181(11.6%)	83/133 (62.4%)	<0.001	−59.6%; −40.8%
Stage of cervical elongation without uterine prolapse				
Stage I	2/21(9.5%)	5/83 (6.0%)	0.012	−8.9%; 19.7%
Stage II	2/21 (9.5%)	37/83 (44.6%)		−46.3%; −9.8%
Stage III	17/21 (81.0%)	41/83 (49.4%)		5.3%; 45.9%
Presence of rectocele	78/181(43.1%)	45/133 (33.8%)	0.113	−1.6%; 19.8%
Stage of rectocele				
Stage I	37/78 (47.4%)	19/45 (42.2%)	0.261	−12.9%; 29.8%
Stage II	19/78 (24.4%)	17/45 (37.8%)		−30.1%; 3.5%
Stage III	22/78 (28.2%)	9/45 (20.0%)		−7.9%; 22.8%
Presence of enterocele	2/181 (1.1%)	1/133 (0.8%)	1	−2.6%; 2.9%
Stage of enterocele				
Stage I	0/2 (0%)	0/1 (0%)	1	−76.5%; 59.8%
Stage II	1/2 (50%)	1/1 (100%)		−89.1%; 55.8%
Stage III	1/2 (50%)	0/1 (0%)		−55.8%; 89.1%

**Table 2:** The sensitivity and specificity of the different types of POP in patients with a definitive diagnosis after clinical examination using the POP-Q system in the presurgical consultation and the sensitivity and specificity of the different types of POP patients with an uncertain diagnosis after clinical examination using the POP-Q system in the presurgical consultation.

	Sensitivity (%)	95% CI	Specificity (%)	95% CI
According to the clinical assessment in patients with a definitive diagnosis after clinical examination using the POP-Q system (*n*: 181)				
Cystocele	97.9%	93.9%; 99.6%	95.1%	83.5%; 99.4%
Uterine prolapse	98.3%	90.8%; 99.9%	100%	97.1%; 100%
Cervical elongation	100%	82.4%; 100%	99.4%	96.6%; 99.9%
Rectocele	92.9%	76.5%; 99.1%	90.2%	84.4%; 99.4%
Enterocele	100%	2.5%; 100%	99.4%	96.9%: 99.9%
According to the clinical assessment in patients with an uncertain diagnosis after clinical examination using the POP-Q system (*n*:133)				
Cystocele	91.7%	83.6%; 96.6%	91.8%	80.4%; 97.7%
Uterine prolapse	40.9%	20.7%; 63.7%	83.8%	75.6%; 91.1%
Cervical elongation	79.5%	69.2%; 87.6%	76.0%	61.8%; 86.9%
Rectocele	88.9%	51.8%; 99.7%	85.5%	78.0%; 91.2%
Enterocele	33.3%	0.8%; 90.6%	100%	97.2%; 100%
According to the ultrasound assessment in patients with an uncertain diagnosis after clinical examination using the POP-Q system (*n*:133)				
Cystocele	97.6%	91.7%; 99.7%	81.6%	68.0%; 91.2%
Uterine prolapse	95.5%	77.2%; 99.9%	99.1%	95.1%; 99.9%
Cervical elongation	92.8%	84.9%; 97.3%	96.0%	86.3%; 99.5%
Rectocele	100%	66.4%; 100%	89.5%	82.7%; 94.3%
Enterocele	100%	29.2%; 100%	98.5%	94.4%; 99.8%

**Table 3:** Shows the improvements in diagnostic capacity of the sequential diagnostic protocol for surgical POP.

	Sensitivity (%) 95% CI	*p*	Specificity (%) 95% CI	*p*
Patients with an uncertain diagnosis after clinical examination using the POP-Q system (*n*:133)				
Cystocele	0.5%; 11.3%	<0.05	−18.3%; −2.1%	<0.05
Uterine prolapse	45.5%; 63.7%	<0.05	8.8%; 21.8%	<0.05
Cervical elongation	5.2%; 21,4%	<0.05	12.0%; 27.9%	<0.05
Rectocele	5.7%; 16.4%	<0.05	3.9%; 11.9%	<0.05
Enterocele	58.7%; 74.7%	<0.05	−3.6%; −0.5%	NS

NS: Not significant.

## Data Availability

Dataset available on request from the authors.

## Abbreviations

POP	Pelvic organ prolapse
POP-Q	Pelvic organ prolapse quantification
BMC	Body mass index
